# Observational study of the recent efficacy and economy of itraconazole vs. voriconazole in perioperative pulmonary aspergillosis

**DOI:** 10.3389/fsurg.2025.1553699

**Published:** 2025-04-07

**Authors:** Peng Chen, Xiao Zhang, Xinzhe Zheng, Yang Tang, Yongxiang Song, Gang Xu, Xixian Ke, Cheng Chen

**Affiliations:** Department of Thoracic Surgery, Affiliated Hospital of Zunyi Medical University, Zunyi, Guizhou, China

**Keywords:** itraconazole, voriconazole, antifungal therapy, pulmonary aspergillosis, perioperative

## Abstract

**Background:**

Antifungal therapy is a common treatment option for pulmonary aspergillosis, but its use to prevent recurrence and relieve symptoms in patients with pulmonary aspergillosis remains controversial. This study aimed to explore the short-term efficacy and cost of itraconazole vs. voriconazole in the treatment of pulmonary aspergillosis during the perioperative period.

**Methods:**

We retrospectively collected the clinical data of 55 patients with pulmonary aspergillosis who underwent surgery and received itraconazole or voriconazole as antifungal therapy between January 1, 2016, and October 31, 2022, at the Department of Thoracic Surgery, Affiliated Hospital of Zunyi Medical University. We analyzed the effects of different antifungal therapies on the incidence of adverse events, short-term efficacy, and cost-effectiveness of pulmonary aspergillosis treatment.

**Results:**

Except for the younger average age of patients in the itraconazole group, there were no significant differences in baseline characteristics such as sex, BMI, underlying lung disease, accompanying symptoms, and lesion location between the voriconazole and itraconazole groups. There was no significant difference in the incidence of adverse events or short-term efficacy, including recovery, symptom improvement, and recurrence rates, during the administration of the antifungal regimens between the two groups. Regarding economic cost efficiency, there was no significant difference in the median hospitalization costs between the two groups. However, despite the longer antifungal treatment duration in the itraconazole group, the cost of itraconazole was significantly lower than that of voriconazole.

**Conclusion:**

Both itraconazole and voriconazole effectively prevented the recurrence of pulmonary aspergillosis. They are similar in terms of the incidence of adverse events and short-term efficacy; however, itraconazole is more economical.

## 1 Introduction

Chronic pulmonary aspergillosis (CPA) poses a significant threat to public health worldwide. Epidemiological surveys show that approximately 3 million individuals suffering from CPA globally, with a mortality rate as high as 40%–50% (1–4). Pulmonary aspergillosis (PA) is a special type of CPA characterized by the accumulation of fungal hyphae, fibrin, mucus, and cell debris in the lung cavity (2). This condition frequently arises as a complication of pre-existing pulmonary ailments, such as tuberculosis, chronic bronchitis, bronchiectasis, and lung cancer (3, 5), among which tuberculosis is the most common precursor in China (6). Widespread use of corticosteroids and broad-spectrum antibiotics has significantly increased the incidence and mortality rates of PA. Despite its severity, PA often exhibits non-specific symptoms, leading to considerable delays in diagnosis and treatment, thus missing the key window for effective intervention.

The diagnosis of PA requires a holistic approach that integrates clinical observations, radiological insights, and corroborative serological and microbiological evidence (5). PA is divided into solitary PA, which is often asymptomatic, and complex PA, with more pronounced symptoms such as coughing and hemoptysis. Approximately 40% of patients with PA experience additional discomfort such as wheezing, weight loss, and fever (7). According to Niu et al., the most prevalent symptoms include hemoptysis (65.2%) and cough (87.0%) (6). These non-specific symptoms lead to the long-term inability to diagnose PA. Computed tomography (CT) is a pivotal diagnostic tool; CT scan of the chest displays aspergillomas in the upper lobe of the lungs, characterized by an “air crescent sign” lacking significant enhancement after injection of contrast agent (8). Uncommon age-related findings may include irregular spongy formations that do not change with the patient's position and may exhibit granular or complete calcification (8). Serological testing and microbiological validation achieved through blood analysis, puncture biopsies, or cultures from bronchoalveolar lavage fluid (BALF) or sputum are the key criteria for diagnosing PA (9, 10). Key assessments encompass the (1,3)-β-D-glucan (BDG) test, galactomannan (GM) assay, fungal cultures, and Aspergillus IgG antibody determinations. The latter is a component of the diagnostic criteria for CPA endorsed by the Infectious Diseases Society of America (IDSA) and the European Society of Clinical Microbiology and Infectious Diseases (ESCMID) in collaboration with the European Respiratory Society (ERS) (11, 12), with a missed diagnosis rate of approximately 10%–12% (13). Although BDG, GM, and fungal cultures can aid in the diagnosis (14, 15), prolonged culture times and low sensitivity are notable limitations. Similarly, the sensitivity of granulocyte cultures from the BALF or sputum does not meet expectations (16). Therefore, the reliance on serological and microbiological testing in clinical settings continues to seek reliable clinical evidence to bolster its effectiveness (11).

When diagnosing PA, treatment options include antifungal medications (both oral and intravenous), surgical measures, and palliative care strategies (17). Oral treatment predominantly employs triazole agents, which have been proven to be effective in approximately 50%–80% of PA cases (18). In clinical practice, itraconazole, voriconazole, and posaconazole are the preferred triazoles drugs (19). As first-line therapies, itraconazole and voriconazole could be interchanged (20–22). Amphotericin B and caspofungin are substitutes for IV administration, especially in infections resistant to triazole compounds (23). The necessity for surgical intervention in asymptomatic individuals with a solitary PA remains a contentious topic (24, 25). However, for patients with local symptoms, antifungal therapy alone is often insufficient, making surgical excision the preferred treatment method (26–28). Recent findings have suggested that surgery has a 5%–7% recurrence rate (29). Although specific clinical guidelines and controlled study results are lacking, administration of antifungal drugs before and after surgery has been identified as an effective measure to prevent postoperative recurrence (30). Gebitekin et al. highlighted that oral itraconazole administered two weeks before surgery and three months after surgery could successfully remove aspergilloma without postoperative death or major complications (31). In contrast, Sagan et al. reported that postoperative adjunctive antifungal treatment does not improve patient outcomes (32). Long-term antifungal therapy can help alleviate symptoms, such as coughing, weight loss, and fatigue (2, 33), with two-thirds of patients experiencing improved quality of life (20, 34). However, a great challenge is that the antifungal therapy has a slow onset and long duration (33, 35), typically lasting 3–6 months. A randomized controlled trial revealed that 75% of patients receiving six-month of itraconazole treatment showed signs of improvement or disease stability, whereas 25% experienced recurrence after discontinuation of the medication (34). Furthermore, the long-term use of antifungal drugs is associated with potential adverse reactions, including liver impairment, peripheral neuropathy, heart failure, and leukopenia, as well as the risk of drug resistance. Therefore, there is an urgent need to determine optimal timing, selection, and duration of antifungal therapies. In this study, we investigated the short-term efficacy and economy of itraconazole and voriconazole for the perioperative antifungal treatment of PA.

## 2 Materials and methods

### 2.1 Ethics statement

The studies involving humans were approved by the study adhered to the ethical standards of the Declaration of Helsinki (2013 revision) and was approved by the Ethics Committee of Affiliated Hospital of Zunyi Medical University. The studies were conducted in accordance with local legislation and institutional requirements. All the participants provided written informed consent to participate in this study. Written informed consent was obtained from each individual(s) for the publication of potentially identifiable images or data included in this article.

### 2.2 Research participants

This retrospective study gathered data from 55 individuals with pulmonary aspergilloma who underwent surgical procedures and were treated solely with itraconazole or voriconazole at the Affiliated Hospital of Zunyi Medical University between January 1, 2016, and October 31, 2022. Based on their specific antifungal therapy regimens, the participants were categorized into the itraconazole or voriconazole groups. This study aimed to evaluate the influence of itraconazole and voriconazole on the postsurgical recovery of patients with PA, and to investigate the immediate effectiveness and financial viability of itraconazole and voriconazole in the management of pulmonary aspergilloma.

### 2.3 Inclusion criteria

(1) Individuals who had received surgical intervention and were pathologically diagnosed with PA. (2) Subjects with a solitary pulmonary aspergilloma lesion were successfully excised and treated exclusively with itraconazole or voriconazole during their antifungal regimen.

### 2.4 Exclusion criteria

(1) Individuals diagnosed with multiple pulmonary aspergillomas where the surgical approach was limited to palliative resection and failed to completely remove all infected areas. (2) Subjects who were administered antifungal treatments other than triazoles during the therapy period or who received a combination of itraconazole and voriconazole. (3) Subjects untraceable during follow-up. (4) Patients who failed to consistently attend follow-up appointments or did not undergo the necessary imaging or serological evaluations during their treatment course. (5) Subjects presenting with uncorrected abnormalities in liver and kidney function or white blood cell counts before initiating antifungal therapy.

### 2.5 Treatment protocol

All patients underwent surgery. For patients suspected of having intraoperative Aspergillus contamination in the thoracic cavity, itraconazole capsules (oral administration, 200 mg per dose, once daily) or voriconazole (intravenous infusion for hospitalized patients and oral voriconazole tablets for discharged patients, 200 mg per dose, twice daily) were administered. During the antifungal treatment period, liver and kidney functions were monitored, and chest CT scans were regularly reviewed. Based on the different antifungal treatment plans, the antifungal + surgery group was further divided into itraconazole and voriconazole groups.

### 2.6 Clinical indicators

We collected the patients’ age, sex, underlying diseases, nutritional status [height, weight, body mass index (BMI)], admission symptoms, blood indicators, surgical methods, duration of surgery, chest CT radiological findings, antifungal adverse events (liver function impairment, kidney function impairment, leukopenia), failure of antifungal treatment (radiological findings and symptoms), and cost of antifungal treatment.

### 2.7 Short-term efficacy assessment

Therapeutic outcomes and incidence rates of antifungal adverse events were comprehensively evaluated. The treatment efficacy was assessed using a composite index of clinical, radiological, and overall clinical improvements. Recovery was defined as the absence of clinical manifestations such as hemoptysis, cough, or expectoration, and radiological examinations showing no recurrence of aspergilloma. Improvement was defined as the alleviation of clinical symptoms, and radiological examinations showed no recurrence of the aspergilloma. Relapse was defined as radiological evidence of aspergilloma recurrence or aggravation of clinical symptoms. The incidence rates of antifungal adverse events, including liver function impairment, kidney function impairment, and leukopenia, during antifungal treatment were calculated. The cost of antifungal treatment was used as an economic evaluation indicator.

### 2.8 Statistical methods

Statistical analyses were conducted using SPSS 25.0. The Mann–Whitney *U* test was used to compare quantitative data between different groups, and the chi-squared test was used to compare categorical data between groups. *p* < 0.05 (if not specified) was considered statistically significant.

## 3 Result

### 3.1 Baseline characteristics of patients

There are 26 patients in the itraconazole cohort with a median age of 48.9 years (39–51) and a median BMI of 22.6 kg/m^2^ (19.9–24.2 kg/m^2^). Among them, 16 were male (61.5%). Tuberculosis was the most prevalent underlying lung disease, identified in 17 patients (65.4%), followed by bronchiectasis in 10 patients (38.5%). With regard to accompanying symptoms, the proportion of patients with hemoptysis was the highest, accounting for 16 cases (61.5%), followed by cough with phlegm in 14 cases (53.9%). These patients had an average white blood cell count of 5.8 (±1.7) × 10^9 ^/L, with an average albumin level of 39.8 (±3.8) g/L. Median pulmonary function metrics included an FEV1 of 2.6 L (1.9–3.1 L) and an MVV of 98.66 L/min (78.8–121.1 L/min). The voriconazole group comprised 29 patients with a median age of 52 years (range, 46–60 years) and median BMI of 21.4 kg/m^2^ (19.9–23.9 kg/m^2^). This group included 19 males (65.5%). Similar to the itraconazole group, tuberculosis was the leading underlying lung disease, identified in 23 patients (79.3%), and bronchiectasis was found in 12 patients (41.4%). Hemoptysis (65.5%) and cough with expectoration (37.9%) were observed in 19 and 11 patients, respectively. The average white blood cell count was 6.1 (±2.5) × 10^9 ^/L, and the average albumin level was 38.8 (±3.8) g/L. Pulmonary function showed a median FEV1 of 2.6 L (2.1–3.1 L) and an MVV of 99.8 L/min (87.3–123.0 L/min). Despite the younger median age in the itraconazole group (*p* = 0.025), no significant differences were found between the groups in terms of BMI, sex distribution, underlying lung conditions, symptomatic presentations, blood metrics, pulmonary function tests, or surgical duration (*p* > 0.050) (Table 1).

**Table 1:** Baseline characteristics the patients.

Variables	Itraconazole group (*n* = 26)	Voriconazole group (*n* = 29)	*p*
Age, years, (IQR)	46 (12)	52 (14)	0.025
BMI, kg/m^2^, (IQR)	21.9 (4.3)	21.4 (4.0)	0.877
Sex, Male, *n* (%)	16 (61.5)	19 (65.5)	0.795
Prevalent underlying lung diseases, *n* (%)			
Pulmonary Tuberculosis	17 (65.4)	23 (79.3)	0.247
Bronchiectasis	10 (38.5)	12 (41.4)	0.825
Frequency of underlying diseases, *n* (%)			
Diabetes	2 (7.7)	2 (6.9)	1.000
Hypertension	5 (19.2)	1 (3.5)	0.090
Common accompanying symptoms, *n* (%)			
Hemoptysis	16 (61.5)	19 (65.5)	0.759
Cough and expectoration	14 (53.9)	11 (37.9)	0.237
Blood parameters			
WBC, ×10^9 ^/L, (SD)	5.8 (1.7)	6.1 (2.5)	0.897
Albumin, g/L, (SD)	39.8 (3.8)	38.8 (3.8)	0.252
Pulmonary function, (IQR)			
FEVI, L	2.6 (1.2)	2.6 (0.9)	0.697
MVV, L/min	98.7 (42.36)	99.8 (35.7)	0.917

Quantitative data are expressed as standard deviation (s) and interquartile range (IQR); SD, standard deviation; IQR, interquartile range; BMI, body mass index; WBC, white blood cell; FEV1, forced expiratory volume in 1 s; MVV, maximal voluntary ventilation; *P*, *P* value.

### 3.2 Comparison of surgical features and aspergilloma position

In the itraconazole group, 18 patients (69.2%) underwent lobectomies and 3 (11.5%) underwent sub-lobar resections, with an average surgical duration of 3.7 ± 1.5 h. Aspergillomas were predominantly located in the upper (*n* = 19) and lower lobes (*n* = 4). Comparatively, in the voriconazole group, lobectomies were performed on 20 patients (69.0%), and 8 had sub-lobar resections, with an average surgical time of 4.0 ± 1.2 h. Aspergillomas were identified in the upper lobes of 22 patients and in the lower lobes of 5 patients. However, there were no significant differences in surgical duration, prevalence of lobectomy, or aspergilloma positioning between the two groups (*p* > 0.050) (Table 2).

**Table 2:** Comparison of surgical features and aspergilloma position.

Variables	Itraconazole group (*n* = 26)	Voriconazole group (*n* = 29)	*p*
Lobectomy, *n* (%)	18 (69.2)	20 (69.0)	0.983
Sub-lobar resection, *n* (%)	3 (11.5)	8 (27.6)	0.137
Duration of surgery, h, (SD)	3.7 (1.5)	4.0 (1.2)	0.825
Location of aspergilloma, *n* (%)			
Upper lung	19 (73.1)	22 (75.9)	0.813
Right middle lung	0	1 (3.5)	–
Lower lung	4 (15.5)	5 (17.2)	1.000

Quantitative data are expressed as standard deviation (s); Count data are presented as frequencies and percentages (%); SD, standard deviation; P, *P* value.

### 3.3 Comparison of adverse event rates

In the itraconazole group, antifungal adverse events occurred in six patients, including impairment of liver function in five patients and leukopenia in one patient. The median liver function index was ALT: 21.0 U/L (15.0–46.5 U/L), AST: 28.5 (20.8–41.5) U/L. The median renal function index was creatinine: 62.0 μmol/L (55.0–76.3 μmol/L). In the voriconazole group, there were four cases of antifungal adverse events, all of which involved liver function damage. The median liver function index was ALT: 14.1 U/L (10.5–30.0 U/L), AST: 29.0 U/L (22.5–46.0 U/L). The median kidney function index was creatinine: 64.0 μmol/L (50.5–73.5 μmol/L). There was no significant difference in the incidence of adverse events between the two groups (*p* > 0.050) (Table 3).

**Table 3:** Incidence of adverse events.

Variables	Itraconazole group (*n* = 26)	Voriconazole group (*n* = 29)	*p*
Antifungal adverse events, *n* (%)	6 (23.1)	4 (13.8)	0.490
Liver function impairment	5 (19.2)	4 (13.8)	0.721
Leukopenia	1 (3.9)	0	–
ALT, U/L, (IQR)	21.0 (31.5)	14.1 (19.5)	0.128
AST, U/L, (IQR)	28.5 (20.8)	29.0 (23.5)	0.970
Creatinine, μmoI/L, (IQR)	62.0 (21.3)	64.0 (23.0)	0.890

Quantitative data are expressed as count data are presented as frequencies and percentages (%); P, *P* value; ALT, alanine aminotransferase; AST, aspartate aminotransferase; IQR, interquartile range.

### 3.4 Comparison of treatment outcomes

In the itraconazole group, 25 patients recovered, with a recovery rate of 96.2%, and one patient showed improvement in symptoms. No aspergillosis recurrence was observed in any patient. In the voriconazole group, 24 patients showed complete resolution of symptoms (recovery rate: 82.75%), and three patients achieved improvement in symptoms. Two patients experienced aspergilloma recurrence. There was no significant difference in the treatment effect between the itraconazole group and voriconazole group (*p* > 0.050) (Table 4).

**Table 4:** Postoperative data.

Variables	Itraconazole group (*n* = 26)	Voriconazole group (*n* = 29)	*p*
Recovery, *n* (%)	25 (96.2)	24 (82.8)	0.197
Free of recurrence, *n* (%)	26 (100)	27 (93.1)	-
Symptom improvement, *n* (%)	1 (3.8)	3 (10.3)	0.613

Count data are presented as frequencies, percentages (%), and *P* values.

### 3.5 Economic analysis

In the itraconazole group, the median duration of antifungal treatment was 22days (IQR = 9 days), with a median cost of antifungal therapy of 770 RMB (IQR = 1,015 RMB) and a median hospitalization expense of 69,480 RMB (IQR = 24426 RMB). In contrast, the voriconazole group experienced a median antifungal treatment duration of 10.00 days (IQR = 8 days), with a median antifungal therapy cost of 2,000 RMB (IQR = 1,500 RMB), and a median hospitalization cost of 66,139 RMB (IQR = 36,500 RMB). No significant difference was observed in hospitalization expenses between the itraconazole and voriconazole groups (*p* > 0.050). However, compared to the voriconazole group, the duration of antifungal treatment in the itraconazole group was significantly longer (*p* < 0.001), and the antifungal treatment cost of the itraconazole group was lower than that of the voriconazole group (*p* < 0.001) (Table 5).

**Table 5:** Economic analysis.

Variables	Itraconazole group (*n* = 26)	Voriconazole group (*n* = 29)	*p*
Duration of antifungal treatment, d, (IQR)	22 (9)	10 (8)	<0.001
Cost of antifungal treatment, RMB (IQR)	770 (1,015)	2,000 (1,500)	<0.001
Costs of hospital stay, RMB, (IQR)	69,480 (24,426)	66,139 (36,500)	0.919

Quantitative data are expressed as interquartile range; IQR, interquartile range; RMB, renminbi; *P*, *P*-value.

## 4 Discussion

Pulmonary aspergilloma (PA), a chronic condition with increasing global incidence (3, 34, 36), frequently eludes timely diagnosis owing to its nonspecific clinical manifestations (20). Current therapeutic strategies exhibit considerable variability, with limited consensus on optimal approaches and a paucity of comparative clinical trial data (37). This study assessed the immediate efficacy and cost-effectiveness of perioperative itraconazole and voriconazole in preventing PA recurrence. Although both antifungals demonstrated comparable short-term efficacy, itraconazole was more cost-effective, supporting its prioritization in perioperative settings.

Pulmonary tuberculosis is a major underlying condition associated with pulmonary aspergilloma, accounting for approximately 15.3%–63% of all cases (38). In our study, we found that the proportion of patients with pulmonary tuberculosis was 72.73%, which was significantly higher than this range. This disparity can be attributed to the high prevalence of tuberculosis in Guizhou Province.

Hemoptysis, a potentially life-threatening complication of PA conditions (24, 39), requires tailored treatment. Surgical resection such as lobectomy is preferred for localized lesions, whereas conservative measures such as embolization and hemostatic medication are reserved for patients who are not candidates for surgery. However, the effectiveness of embolization is limited (40–42), with postoperative hemoptysis recurrence rates ranging from 25% to 30% in patients with chronic PA (26). In our cohort, 31 of 35 patients with hemoptysis fully recovered after surgery, and only four cases (11.43%) experienced recurrence, characterized by trace blood in the sputum. These findings highlight the therapeutic value of this surgery. Therefore, surgery may provide more benefits to patients when local lesions are treated.

Our study found no significant differences in BMI, underlying lung conditions, accompanying symptoms, blood markers, pulmonary function, or surgical time between the two groups. However, the median age of the itraconazole group was lower than that of the voriconazole group, which may reflect a selection bias in prioritizing voriconazole in elderly patients.

For patients with symptomatic localized PA, antifungal monotherapy is often insufficient, and surgical resection remains the preferred intervention (26–28). Lobectomy is the standard approach (20, 24, 30, 43–46), Some studies have indicated that sublobar resection may result in residual Aspergillus in the lung tissues, which can increase the risk of postoperative bronchial stump air leaks (30).

Regarding the short-term and long-term prognosis of surgical treatment for PA, studies have shown overall survival rates at 2 years, 5 years, and 10 years to be 86.6%, 79.4%, and 79.4%, respectively, with disease-free survival rates at 86.6%, 72.6%, and 72.6%, respectively (47). The success of the surgery depends on the complete removal of the aspergilloma and the absence of aspergilloma rupture contaminating the thoracic cavity during the surgical process (12, 38, 48). If there is no aspergilloma rupture or spillage of Aspergillus, postoperative antifungal drugs may not be necessary (12, 49).

Current research indicates a 5%–7% recurrence rate of PA in patients undergoing surgery (29). Antifungal drugs are believed to prevent postoperative recurrence, and triazoles are currently the preferred option (11). Studies have shown that the long-term use of triazole drugs can significantly improve symptoms and enhance the quality of life of patients not undergoing surgery (2). However, long-term use of triazole drugs is associated with common adverse events, including peripheral neuropathy, heart failure, elevated transaminase levels, QT prolongation, and photosensitivity (20, 21, 50).

In this study, perioperative patients were administered itraconazole or voriconazole to prevent postoperative recurrence of pulmonary aspergilloma (PA). Follow-up outside the hospital revealed that 49 patients recovered, but 2 cases of recurrence occurred in the voriconazole group, with a recurrence rate of 3.63%, which is lower than the currently reported rate of 5%–7% (29). Thus, the results of this study prove that antifungal medication can effectively prevent the postoperative recurrence of pulmonary aspergilloma. However, there were no significant differences in the recovery, recurrence, or symptom improvement rates between the two groups in this study.

A meta-analysis on long-term oral tolerance to itraconazole and voriconazole showed an adverse event rate of 20%–52% for voriconazole and 18%–31% for itraconazole (21). In this study, the adverse event rates in the itraconazole and voriconazole groups were 23.1% and 13.8%, respectively. The adverse events caused by triazole drugs mainly include liver damage, fatigue, and nausea, and liver function returned to normal after treatment. A more severe adverse event was leukopenia; however, white blood cell levels returned to normal after discontinuation of the medication, and there was no significant difference in the incidence of adverse events between the two groups. Therefore, there were no significant differences in efficacy between itraconazole and voriconazole.

Thus far, only a few clinical studies have evaluated the cost-effectiveness of voriconazole and itraconazole. Findings from this study revealed that although the itraconazole group underwent a longer duration of antifungal treatment than the voriconazole group, the associated costs for itraconazole treatment were lower, suggesting that itraconazole is a more cost-effective option.

In this study, 2 cases of PA recurrence were observed in the voriconazole group. The first case involved a patient who, despite lacking obvious symptoms, showed proliferative lesions on the same side of the lung on chest CT. The second case involved a chest CT scan performed three months after antifungal therapy, which revealed that the upper right lung cavity was full of aspergilloma. These recurrences were attributed to the impaired nutritional status of the two patients, combined with post-operative financial constraints, which hindered adherence to the prescribed regular antifungal therapy, leading to an incomplete treatment regimen.

However, this study has two potential limitations. First, as a single-center retrospective cohort study, it was inevitably subject to selection bias, which limited the generalizability of our findings. Second, the sample size was small and more data or multi-center studies should be included in the future to verify our findings.

## 5 Conclusion

Itraconazole and voriconazole are effective in preventing pulmonary aspergillosis recurrence. There was no difference in the incidence of adverse events or short-term efficacy between the two drugs; however, itraconazole was found to be more economical.

## Data Availability

The original contributions presented in the study are included in the article/Supplementary Material, further inquiries can be directed to the corresponding authors.
